# What drives population fluctuations of European ground squirrels in Hungary?

**DOI:** 10.1186/s12983-026-00608-3

**Published:** 2026-04-08

**Authors:** Csongor István Gedeon, Olivér Váczi, Felix Knauer, Mátyás Árvai, Franz Suchentrunk

**Affiliations:** 1 Department of Soil Mapping and Environmental Informatics, Institute for Soil Sciences, HUN-REN Centre for Agricultural Research, Fehérvári Street 132-144. H-1116, Budapest, Hungary; 2 Herman Ottó Institute Non-Profit Ltd., Park u. 2. 1223, Budapest, Hungary; 3 Research Institute of Wildlife Ecology, University of Veterinary Medicine Vienna, Savoyenstrasse 1, A-1160 Vienna, Austria

**Keywords:** Population density, Spline, Forecast, Geographic location, Ambient winter temperature, Precipitation, Flooding, Aridification

## Abstract

**Background:**

Understanding and predicting the dynamics of rodent populations, particularly for endangered or pest species, requires knowledge of potential background factors. We collected long-term data on the abundance of European ground squirrels, *Spermophilus citellus*, in 64 colonies as part of the Hungarian Biodiversity Monitoring System to investigate the abiotic factors that could explain the spatial and temporal dynamics of populations in Hungary. We used information theory-based modelling and multi-model inference to investigate the effects of environmental and climate variables on relative population densities, using monitoring records of burrow counts as proxies for density collected annually between 2000 and 2018. We examined data carefully for any clues of inconsistencies, errors, or missing data, which resulted in 57% of the complete dataset.

**Results:**

Our generalised additive models (GAM) with splines identified geographical location, year, and principal components reflecting winter temperature, precipitation, and summer temperature to affect population densities. Contour plots derived from the best GAM model uncovered increasing density in the Kiskunság and Kisalföld meso-regions with favourable water management characteristics and decreasing densities in the northern and southern regions, which in fact, cover floodplain of River Tisza and other flash-flood regions. Ambient temperature and precipitation of the wettest and warmest months during hibernation and summer contributed most to counts fluctuations, though these factors were dwarfed by spatial effects. Increased temperatures and aridification in the Kiskunság, because of the warming Pannonian ecoregion, seemed to positively affect counts.

**Conclusions:**

Analysis of our reduced dataset indicated unidentified local factors or a spatial effect on demographic variability of ground squirrel populations. Those fluctuations underscore the necessity of research and management on local populations to identify the reasons of declines and adapt management accordingly to stop or change decreasing population trajectories.

**Supplementary Information:**

The online version contains supplementary material available at 10.1186/s12983-026-00608-3.

## Background

Burrowing mammals are widespread [1, 2], and their bio-perturbation and ecosystem-engineering change the soil characteristics and resources for other organisms [3–5]. Consequently, they are important for maintaining ecosystem functions and services, including human health and well-being [6, 7]. Their disappearance is one of the main reasons for the significant deterioration of natural grassland ecosystems and the loss of related functions and services [8–11]. However, owing to their near-surface habitat, variable activity patterns, and pronounced, sudden population fluctuations, their population density dynamics are less predictable [12, 13], and their conservation management often falls behind.

Potential factors responsible for the frequent fluctuations and negative trends in their populations may include anthropogenic, environmental, and both density-dependent and independent factors, including intrinsic, self-regulatory mechanisms with physiological and behavioural responses to stress [14]. More specifically, unfavourable habitat management methods, changes in land use, alternating productivity (quantity and quality of food) of habitats or density of predators, and changes in mortality rates (of females and juveniles particularly) due to various reasons could result in fluctuating abundance of their populations [15–17]. There are other density-independent environmental factors, such as shifted weather patterns, increasing temperatures, and changing precipitation patterns, that are expected to become more influential on the reproduction and survival of small mammals as climate change and other anthropogenic factors prevail globally. In the Carpathian Basin, where three endangered characteristic burrowing rodent species live [18], the European ground squirrel (*Spermophilus citellus*, hereafter, EGS) [19–21], and two species of the Lesser blind molerat species complex (*Nannospalax hungaricus* and *N. montanosyrmiensis*) [22], those environmental factors will become even more influential.

Global warming is one of the obvious potential reasons for intensified fluctuations or a decline in rodent populations in certain localities [15], and hibernating species are particularly vulnerable to warming winters in temperate climates [23]. Specifically, warming winters of temperate climates are expected to influence the distribution range and abundance of rodents, and within the Carpathian Basin even more intensively because of the increased temperature and intensified precipitation changes in this region, probably due to its geographic position at the intersection of three climatic zones, namely the Continental steppe, Mediterranean, and Oceanic (Atlantic) climates. Climate models predict that the spatial pattern of precipitation by the mid and end of the twenty-first century will not change remarkably, but the temporal distribution will reorganise, resulting in dryer summer and wetter autumn/winter periods [24], and an average (median) 1.3 (SD = 0.3) °C temperature increase monthly, seasonally, and annually [25, 26]. These weather changes could significantly affect the survival of hibernating and burrowing mammals during both hibernation and active periods [27–29] or through indirect mechanisms, such as introduced alien species, vegetation changes, or more comprehensive ecosystem changes in this area. Other climate model simulations using the representative concentration pathways of CO_2_ emission scenarios also agree on the general trend in precipitation, a likely decrease in summer and an increase during winter months [24, 28]. All these forecast scenarios indicate that the associated altered weather patterns may act as important drivers of population fluctuations. However, it is often difficult to identify the relevant smaller-scale proximal factors or mechanisms explaining the large fluctuations and (regional) extinctions of animal species. For burrowing hibernating mammals, such as ground squirrels, which will live under more unpredictable conditions due to climate related factors, it seems an even more challenging task [30].

Among the three endangered burrowing rodent species, EGS is decreasing, but still widespread in the Carpathian Basin. It is a medium-sized, soil-inhabiting species found in European shortgrass steppes (Pannonian ecoregion). Its main natural habitats are the Pannonic loess and sand steppes dominated by fescue species [31–33]; however, grassy airfields, golf courses, vineyards, and other agricultural areas such as meadows have become refuges for the species. Previously, it was considered a pest, then it became vulnerable, and later an endangered species in 2020 [21, 34], which intuitively illustrates that its population trajectory has shown a negative trend in many parts of its distribution range in Europe. Long-term data in Hungary have suggested a declining population trend with breakpoints and sudden extinctions or fluctuations in population density in individual colonies [35]. Moreover, these density fluctuations seem to occur asynchronously, but this hypothesis has not yet been supported [31, 36–39]. EGS hibernate for approximately 7 months or stay in their underground burrows during inactive periods, but they feed on the surface and do not cache food [31]. Burrows go into the soil at an angle of 90 or 25–30 degrees (with mounds at the entrances around the latter) against the horizontal surface [40–43] up to a depth of 2 m. Few excavations in Hungary have shown that nests are around the depth of the frost line (approximately 80 cm). Burrow mounds and openings are visible for a long time on the surface if vegetation does not cover them [44].

The European ground squirrel is subject to mandatory monitoring under the Habitats Directive (92/43/EEC) in countries where populations exist. It is listed in Annexes II and IV of the Directive, meaning that Member States must both designate protected areas and regularly monitor its conservation status(5, 45). Visual surveys of specimens or counting active burrow openings are the most common methods. The solitary lifestyle and burrow dwelling of adult specimens provide a way of estimating their relative density and area of occupancy [43, 46–48] non-destructively by counting the density and spatial coverage of active (used) burrows. Overall, the number of active burrows shows a strong correlation with actual density [37, 49–51], though the relationship can depend on density, such as for the common vole (*Microtus arvalis*) [52].

Their abundance has been surveyed in various European countries, such as Austria, Bulgaria, Czech Republic, Poland, Serbia, and Slovakia, for several years [53]. In Hungary, state nature conservation has been carrying out burrow counts since 2000 [39, 54–56].This method uses the density of active burrow openings, counts, as an index of population density. There are both benefits and pitfalls of this technique, including a simple, non-destructive, well-defined or standardised protocol to count only the burrow occupation of potentially breeding adults of a colony [57] (in April, only those are actively present in the colonies); however, it is a proxy index and, as a result, it is inappropriate for estimating the actual, absolute abundance of animals in a colony, which means that it cannot be directly translated into numerical abundance [16, 37, 50, 51, 58]. Nonetheless, the fluctuations in the counts can still show a temporal pattern or trend and can be used to compare densities between years and places monitored at approximately the same period of every year and to study the changes in population density temporally and spatially. Owing to the life history of EGS (i.e. hibernation for seven months and one litter annually), counting active burrows once annually as an index of the density of breeding adults seems an acceptable compromise for density estimations of their populations.

We aimed to investigate if we could identify the abiotic factors underlying recent fluctuations and declines in the relative abundance of Hungarian EGS colonies [35, 39]. Moreover, we were interested in whether there was any evidence of regular synchronised changes in counts over larger regions or indication to population cycles. To answer these questions, we used a time series of yearly counts. Abiotic factors in this study included habitat management and weather parameters, though we are aware of other potentially important factors, such as soil characteristics, that may contribute to their survival significantly. Preliminarily, we defined potential variables within each factor that were expected to affect the population dynamics of local colonies of EGS. These variables encompass management methods, since they prefer short or medium-tall grass [43], therefore their legal protection and proper management (i.e. mowing or grazing) of their habitats are also expected to affect their survival. Variability of ambient temperature and precipitation was also included, given its potential to substantially affect population density [59]. For instance, elevated temperatures during the hibernation period could trigger more frequent and longer arousal periods during hibernation which could decrease the body weight and fitness of animals [27], or more intensive precipitation periods during summer could result in local flash floods or a higher water table that may kill or deteriorate the health of animals, in addition to parasitic infections due to unfavourable weather conditions [60]. Several hypotheses could be formulated based on the variation in temperature and precipitation that might have influenced the fitness, survival, or reproduction of animals. All these weather-linked parameters would eventually affect the density of colonies, represented by counts. As a result, we aimed to narrow down the available variables to a few parameters that could explain the fluctuation of EGS populations in Hungary, which would help strengthen evidence-based planning of conservation efforts related to EGS.

## Methods

### Study sites

**Fig. 1:**
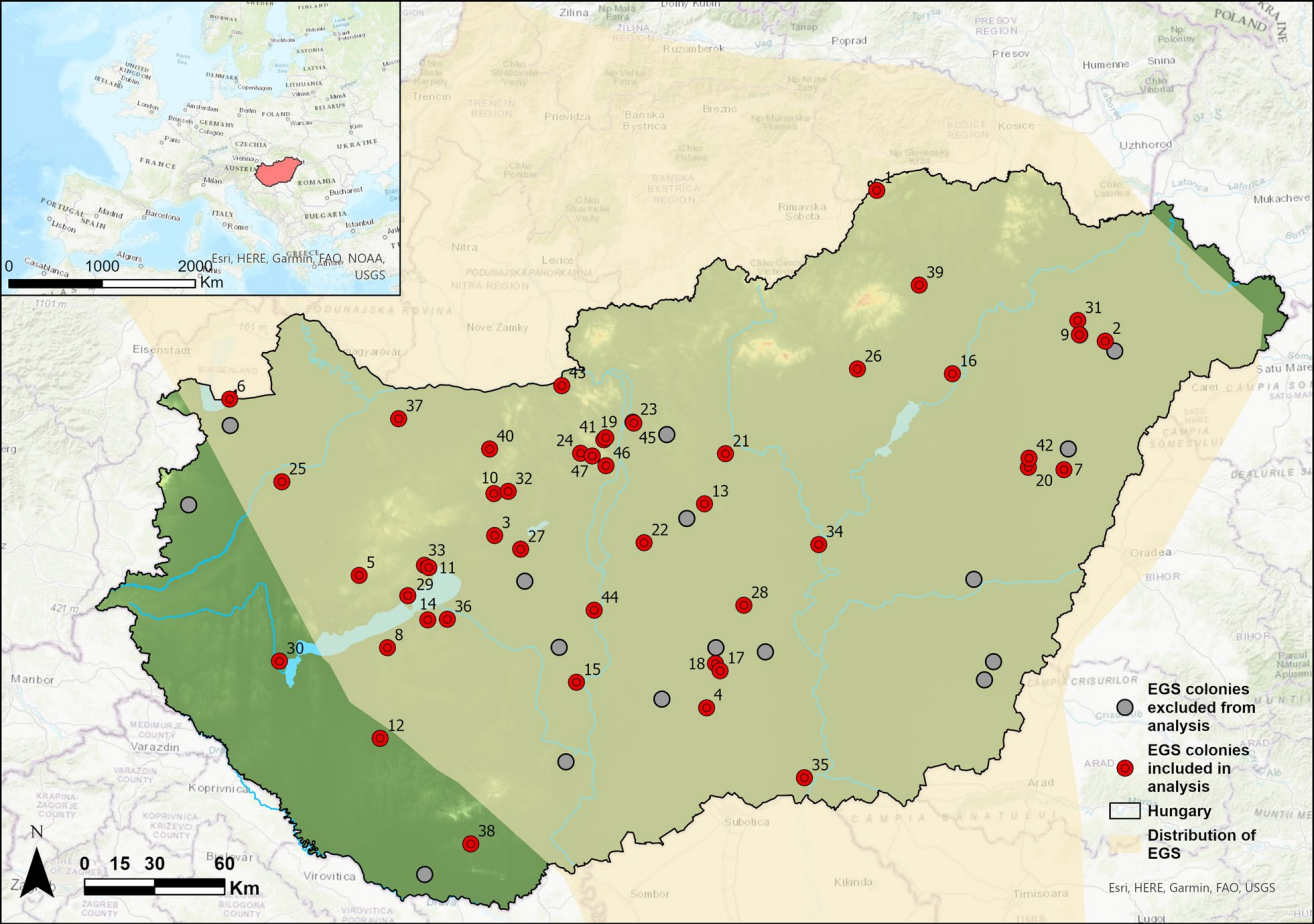
The distribution of ground squirrel colonies in Hungary included in this study. The pale-yellow area shows the northern part of the species’ distribution range registered in the distribution map of IUCN Red List of the EGS [22], in which the Pannonian ecoregion and Hungary within it cover its central area (Legend of red and grey dots is found on the figure). Numbers correspond to colony IDs on Supplementary Figure 1

We collected data on the number of actively used burrows (counts) of EGS colonies in Hungary between 2000 and 2018. The data collected and used in further analysis were obtained from the national biodiversity monitoring of EGS in Hungary, the Hungarian Biodiversity Monitoring System [56, 57, 61]. The study initially encompassed 64 sites (colonies or local populations), where synchronous counts were conducted once a year within the week of April 22nd as part of the regular, annual monitoring scheme. However, owing to the absence or inconsistency of data for specific sites and years, 47 sites and 57% of that complete dataset remained in the dataset for analysis. The study sites and corresponding colonies were spatially representative of all EGS colonies in Hungary [62] (Fig. 1).

The selection of these permanent sites occurred in the 1990s, before the EGS monitoring protocol was initiated [54].

The general climate of the study sites and much of Hungary is characterised by a continental climate with warm or hot summers and most precipitation during the warm period. Nevertheless, summer droughts are frequent in grassland habitats, which cover most of the country (average elevation is under 200 m asl) and serve as a habitat for EGS [63]. The mean annual temperature during the period analysed was approximately 12 °C. The mean annual precipitation during this period was between 500 and 750 mm, with a decreasing trend along the southwest-central longitudinal gradient. As a result of the dominance of the continental zone, there is a high variability in temperature and precipitation in summer [64, 65]. This variation has become more intensive due to global climate change [24]. Therefore, location-specific weather data on temperature and precipitation provided data on the variance of weather parameters at the study sites.

### Weather data

Weather data were downloaded from the FORESEE database (http://nimbus.elte.hu/FORESEE/index.html, accessed on 2 August 2019) for the period between 1999 and 2018 for each colony site [65–67]. FORESEE is an open-access meteorological database that covers the 1951–2000 period and contains gridded, observed, and projected daily 2-m ambient temperature and precipitation data for Central Europe. Original weather data were processed before merging with density data for final modelling and analysis. This included defining and calculating different weather variables from daily mean values. We divided the weather data into two temporal periods following the activity and hibernation periods of EGS (September-March, winter data (W); April-August, summer data (S)) as these were expected to affect survival differently in two distinct physiological states (active and inactive/hibernation periods). We calculated the following location-specific variables for each year: *mtss* and *mt24h* (mean monthly temperature between sunrise and sunset, and between 0–24 h), *tp* (total monthly precipitation), *mthm* (mean monthly temperature of the warmest month), *tphm* (total monthly precipitation of the warmest month), m*tcm* (mean monthly temperature of the coldest month), *tpcm* (total monthly precipitation of the coldest month), *mtwm* (mean monthly temperature of the wettest month). Finally, this resulted in a total of 16 variables, eight each for the summer (active) and winter (hibernation) periods. We expected that these variables could affect the survival of EGS during the active or hibernation periods.

### Density data

The Hungarian Biodiversity Monitoring System provided a country wide, quantitative data on the relative density of EGS in the form of counts of active burrows at the specific locations (colonies) between 2000 and 2018. According to the protocol, surveyors count the number of active (used) burrows along a transect line (five times 50 m) within 2 m (one metre to left and right) [39, 57]. That number was converted into counts per hectare for easier interpretation.

### Statistical analysis

Due to a nonlinear (spline), curved pattern of counts of 19 years and across 64 [47] locations and the uneven time spacing (gaps) between sampling, we used a generalised additive model with penalised smoothing splines (GAM) [68, 69] to model burrow counts. Moreover, due to missing and ‘zero’ (no-animal) data from different, consecutive years and colonies the complete dataset contained 415 lines (data entry, n = 415) including 47 colonies. The GAM’s advantage is its flexibility and robustness for modelling ecological relationships by accounting for potential nonlinear trends between dependent and independent (environmental) variables. Moreover, it enables the definition of a direct relationship between each predictor and the dependent variable using smoothing functions [70–72]. Another benefit of GAM and other regression type models, such as linear models, GLMs, and mixed models, lies in its additive structure that helps explain the effect of each predictor on the response variable. These models allow us to evaluate a predictor’s effect on the response variable, while holding the remaining variables constant. This additive structure makes its interpretation more straightforward than of more complex interaction-filled models. Penalised splines could better balance flexibility and smoothness (“wiggliness”) of the model and avoid overfitting [72, 73].

The reduced dataset (theoretical maximum was 893) was the result of a preliminary, careful data control, which aimed to exclude questionable data from the dataset. Data has been collected by national parks, and due to lack of personnel they have not always been able to carry out the monitoring according to the protocol described between 2000 and 2018. To return to the variables, we tried to avoid collinearity by consolidating the number of weather parameters (8 + 8 variables for Winter (hibernating)—Summer (active) periods) by a Principal Component Analyis (PCA). PCA reduced the dimensionality of both variable sets. For the Summer dataset, the eigenvalue distribution indicated that the first three principal components captured most of the variation, together explaining approximately 80% of the total variance (PC1: ~ 42%, PC2: ~ 23%, PC3: ~ 15%). For the Winter dataset, the first three components similarly accounted for 84% of the variance (PC1: ~ 36%, PC2: ~ 32%, PC3: ~ 17%). In both cases, the number of retained components was determined based on the eigenvalue plot. The inflection point was determined (visually), which showed a drop after the third component. We considered those components for the set of GAM spline models as ‘weather’ predictors (S stands for summer or active period, W stands for winter or hibernation; *C1S, C2S, C3S* and *C1W, C2W, C3W*). Principal component loadings with an absolute value ≥ 0.6 were considered to indicate stronger association and helped to explain the components (Table 1).

**Table 1:** Factor loadings of 8 + 8 weather variables on the first three principal components extracted from PCA. Italicised values indicate high loadings (|loading|≥ 0.6). The acronyms stand for the following: W, winter (hibernation); S, summer (active period); *mtss*, mean monthly temperature between sunrise and sunset (℃); *mt24h*, mean monthly temp between 0–24 h (℃); *tp*, total monthly precipitation (mm); *mthm*, mean monthly temperature of the warmest month (℃); *tphm*, total monthly precipitation of the warmest month (mm); *mtcm*, mean monthly temperature of the coldest month (℃), *tpcm*, total monthly precipitation of the coldest month (mm); *mtwm*, mean monthly temperature of the wettest month (℃)

	Coefficient of PC1 (C1W/S	Coefficient of PC2 (C2W/S)	Coefficient of PC3 (C3W/S)
mtssW	*0.96*	− 0.22	0.06
mt24hW	*0.96*	− 0.20	0.08
tpW	0.27	*0.88*	− 0.24
mthmW	0.32	− 0.36	*− 0.69*
tphmW	0.18	*0.89*	0.12
mtcmW	*0.85*	− 0.13	0.17
tpcmW	0.25	*0.81*	− 0.34
mtwmW	0.09	0.23	0.*82*
mtssS	*0.93*	− 0.28	0.04
mt24hS	*0.93*	− 0.33	0.02
tpS	− 0.48	*− 0.83*	0.03
mthmS	*0.78*	− 0.36	− 0.05
tphmS	− 0.46	− 0.60	0.46
mtcmS	0.47	− 0.32	− 0.39
tpcmS	− 0.52	− 0.61	− 0.26
mtwmS	0.31	0.02	*0.87*

As shown in Table 1, C1W was most strongly associated with *mtssW*, *mt24hW, mtcmW* (loading ≥ 0.9), suggesting the effect of mean monthly temperature in winter. C2W showed high positive loading for *tpW*, *tphmW, tpcmW* (loading ≥ 0.8), indicating the effect of monthly precipitation. C3W was characterised by strong associations with *mthmW* and *mtwmW* (|loading|> 0.6), indicating the inverse effect of monthly temperature of the warmest and wettest months. C1S showed show strong association with variables *mtssS*, *mt24hS*, *mthmsS* (loading ≥ 0.7), indicating the effect of mean monthly temperature in summer and the warmest month. *C2S* showed strong negative association with *tpS* (|loading|= 0.83), indicating the effect of total monthly precipitation, and *C3S* showed high positive loading for *mtwmS* (loading = 0.87), indicating the effect of mean monthly temperature of the wettest month.

Finally, the spatial and temporal fluctuations of burrow counts were modelled by 6 weather components *(C1W, C2W, C3W, C1S, C2S, C3S)*, location (*eovx and eovy coordinates*), year of count (*year*), Colony ID (*loccode*), and airfield status of the site (*Airfield*; i.e. a land use type, where its function provides special management providing permanent protection and habitat management, as known as short grass; a categorical variable, a habitat is either an airfield or not). As a rule of thumb, we followed the approach of parsimony and tried to identify the simplest model(s) with the most explanatory power. For that simplicity and interpretability, we did not include any interactions of the predictors in the global model. Moreover, collinearity of smooth terms, also known as concurvity, was checked during modelling.

The initial model showed a violation of model assumptions (i.e. identity link function and the Gaussian error with mean zero and a constant variance), indicating the need for transformation of the response variable. We applied the Box-Cox transformation using the *boxcox*() function from the library MASS [74]. Box-Cox transformation can stabilise variance (reduce heteroscedasticity), make the residuals’ distribution closer to normal, improve splines’ behaviour (by decreasing over and underfitting) and model convergence. Eventually, our global GAM model contained *count_tr*, which was our transformed response variable ‘counts’, and all predictors, i.e., all smoothed (s prefix stands for spline) explanatory variables and the factor *Airfield*:

$$\begin{array}{ll} [m = & GAM(count\_tr\sim s\left( {year} \right) + s\left( {eovy,eovx} \right) \\ & + s\left( {C1S} \right) + s\left( {C2S} \right) + s\left( {C3S} \right) + s\left( {C1W} \right) \\ & + s\left( {C2W} \right) + s\left( {C3W} \right) + Airfield \\ & + s\left( {loccode,bs = re} \right),data = data)]) \\ \end{array}$$

The transformation decreased the value of the generalised cross-validation (GCV) score (the residual sums of squares) [75] in other words, it resulted in a better smoothing parameter lambda, λ. The GCV score serves as a tool to choose the smallest smoothing parameter, λ, and better fitting model. It is designed to minimise the mean squared error term. λ controls the penalty for wiggliness or over-fitting of the model. Other features in the model were: ‘*s*’, the spline function; *C1S, C2S, C3S* and *C1W, C2W, C3W*, the weather components (S for summer and W for winter); *Airfield*; *loccode* (Colony ID) was an individual colony code, a random factor in the model. (As a result, our modelling could rather be considered a GAMM model in the GAM modelling framework, however, for simplicity, we used the GAM term in the text.). In other words, we included the categorical site identifier (1–47) as a random effect using a spline term with a random effects basis, *s(loccode, bs* = *"re")* to account for site level variation. This allowed the model to capture unobserved heterogeneity among sites while avoiding the overparameterisation that would result from treating all 47 colony IDs as fixed effects. To assess the influence of various predictors on counts, we applied two complementary model selection procedures, a more traditional approach of a sequential model selection and an information-theoretic approach identifying a set of multiple, equally probable models. We present both approaches because they are not mutually exclusive, but they rather overlap in the predictors they highlight and together offer a more nuanced understanding of model structure. The traditional procedure shows which model is selected under a single‑best(final)‑model approach, while the information‑theoretic approach (using model ranking and model averaging [73, 76, 77]) reveals the range or set of plausible models. Showing both results and perspectives increases the results’ transparency and strengthens inference by demonstrating which predictors remains influential across multiple, well‑supported models. That helps discuss the findings more comprehensively and put them into a conservation focused framework.

All data analysis and specifically the GAM spline analysis including data transformation, model checking and selection, and model ranking were performed using R statistical software (R Development Core Team, R Foundation for Statistical Computing, Vienna, AT, http://www.R-project.org). For instance, to generate GAM models with different combinations of predictors, we used the *dredge*() function. Theoretically, in case of *k* predictors, dredge generates 2^* k*^ number of models, however, models were ranked based on their conditional AICc values, weight (w), and delta (Δ_i_) values (the difference between the model’s AICc and the minimum AICc among all models), and from a total of 512 models, the five best models will be shown in section Results based on criteria detailed in this section. Conditional AICc extends the traditional AIC framework by incorporating the uncertainty associated with both fixed and random effects. Unlike standard AIC, which evaluates only the fixed effects component, it provides an appropriate penalty for a higher complexity (by random effects and correlation structures). As a result, its usage is recommended for ranking additive mixed models and selecting the most parsimonious models in a hierarchical modelling context. As recommended in the literature, models with Δ_i_ ≤  ~ 2 were considered adequate models (i.e., models with no clearly different probability) and eventually ranked by the number of predictors and their cumulative weights in models (more cumulative weight and fewer predictors are favoured) in addition to the ranking of AICc values from the lowest to the highest. Calculation of cumulative weight is basically model averaging with the aim of evaluating several models and predictors with similar performance [77]. The sum of weights (SW), an indicator of an individual predictor’s effect on the response variable when considering all models, was calculated by the function *sw*().

Regarding the more traditional, sequential model selection [77] procedure, we followed the recommendations of Lai et al. [78] in evaluating the best, final model’s overall performance and relative importance of predictors in that model. Relative variable importance values above 0.7 are considered indicating statistically meaningful effects [76]. For that purpose, we used the *gam.hp*() and *model.avg*() packages. We also used other packages, such as *nlme, mgcv*, *MuMIn*, *gamlss*, and *MASS,* during the analysis [69, 79, 80]. (To support transparent and reproducible implementation of our analytical framework, the R code underlying all analyses is attached in a separate supplementary file (Supplementary Codes 1). We advise the readers/users to review and apply the scripts/codes with care and concentration, as overlooked errors may lead to unintended or altered analytical outcomes.)

## Results

The traditional model selection procedure identified a single best‑fitting, final GAM model (n = 415) which had a R^2^adj of 0.45 and GCV of 30.464. We found that *eovx* and *eovy* were highly significant predictors of counts (*s*(*eovy, eovx*), *p* < 0.001), as was year (*s*(*year*), *p* = 0.002). Several additional predictors showed near‑significant effects, including *Airfield* (p = 0.050), *loccode* (*s*(*loccode*), *p* = 0.046), and the climatic variables *C2W* and *C3W* (*s*(*C2W*), *p* = 0.051; *s*(*C3W*), *p* = 0.091). Other predictors did not show any significant effect on counts. Statistically significant, individual predictors’ contribution to the total adjusted R^2^ explained by this model was between ~ 1% (*s(loccode)*) and ~ 83% (s*(eovx, eovyy)*) (1.42%, *s(C2W)*, 0.06%, *s(C3W)*. Adjusted R^2^ of smooth term *eovy*, *eovx* in a single variable model was 0.405, while other predictors’ adjusted R^2^ was in the range of 0.003 and 0.107 (Supplementary Fig. 2).

**Fig. 2:**
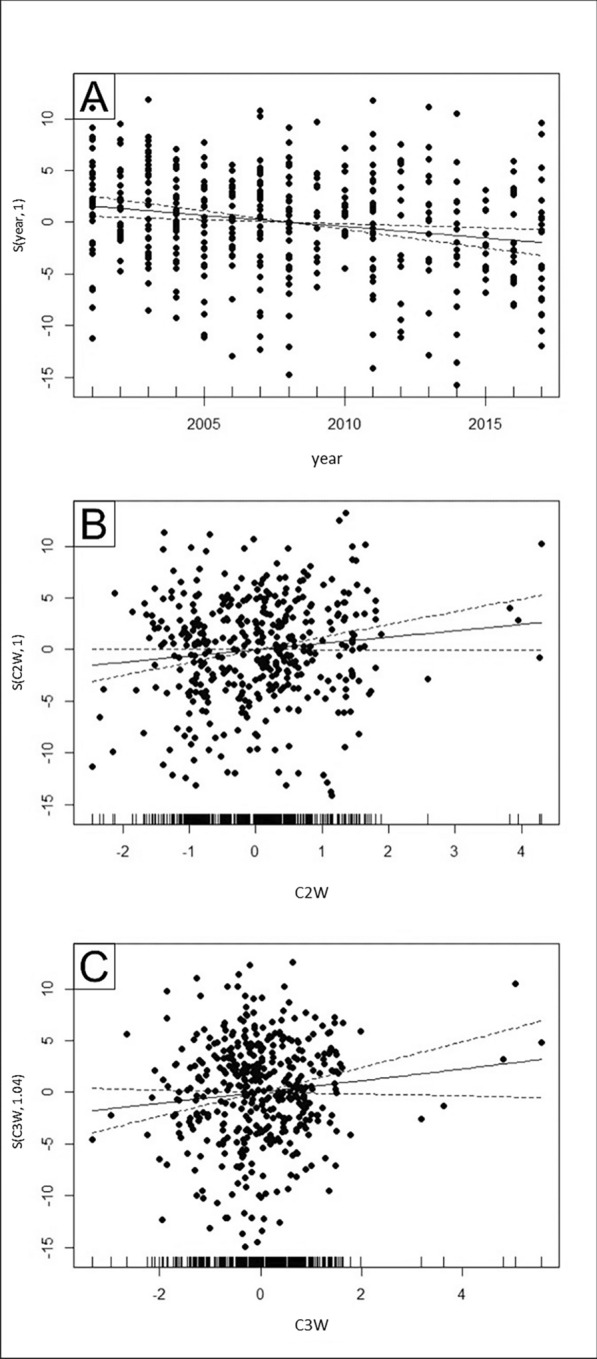
Smooth functions of year versus count showed a small but steady decrease (**A**). Smooth functions of C2W and C3W versus count showed an increasing trend independently of either positive or negative changes of those climatic factors (**B**, **C**)

The smooth functions of *year* versus counts showed a small but steady decrease (Fig. 2 A). Smooth functions of *C2W* and *C3W* versus counts showed an increasing trend independently from either positive or negative changes of those climatic factors (Fig. 2 B, C). For variable *loccode*, the smooth function did not show any trend and kept a constant value (not shown on Fig. 2 A, B, C). Though predictors *year*, *C2W*, *C3W*, and unimportant ones, such as *C1S*, *C2S*, *C3S* indicated a linear response (EDF ~ 1), other predictors, such as *C1W* (EDF ~ 3), *eovx and eovy* (EDF ~ 27) indicated non-linear responses. Consequently, the application of GAM was supported. In addition to the above-mentioned, distribution of data points on Fig. 2A suggested some sinusoidal pattern despite it was not captured by this model. This suggested that not all non-linear patterns might have been captured by GAM consequently it still was favoured approach to the dataset.

**Fig. 3:**
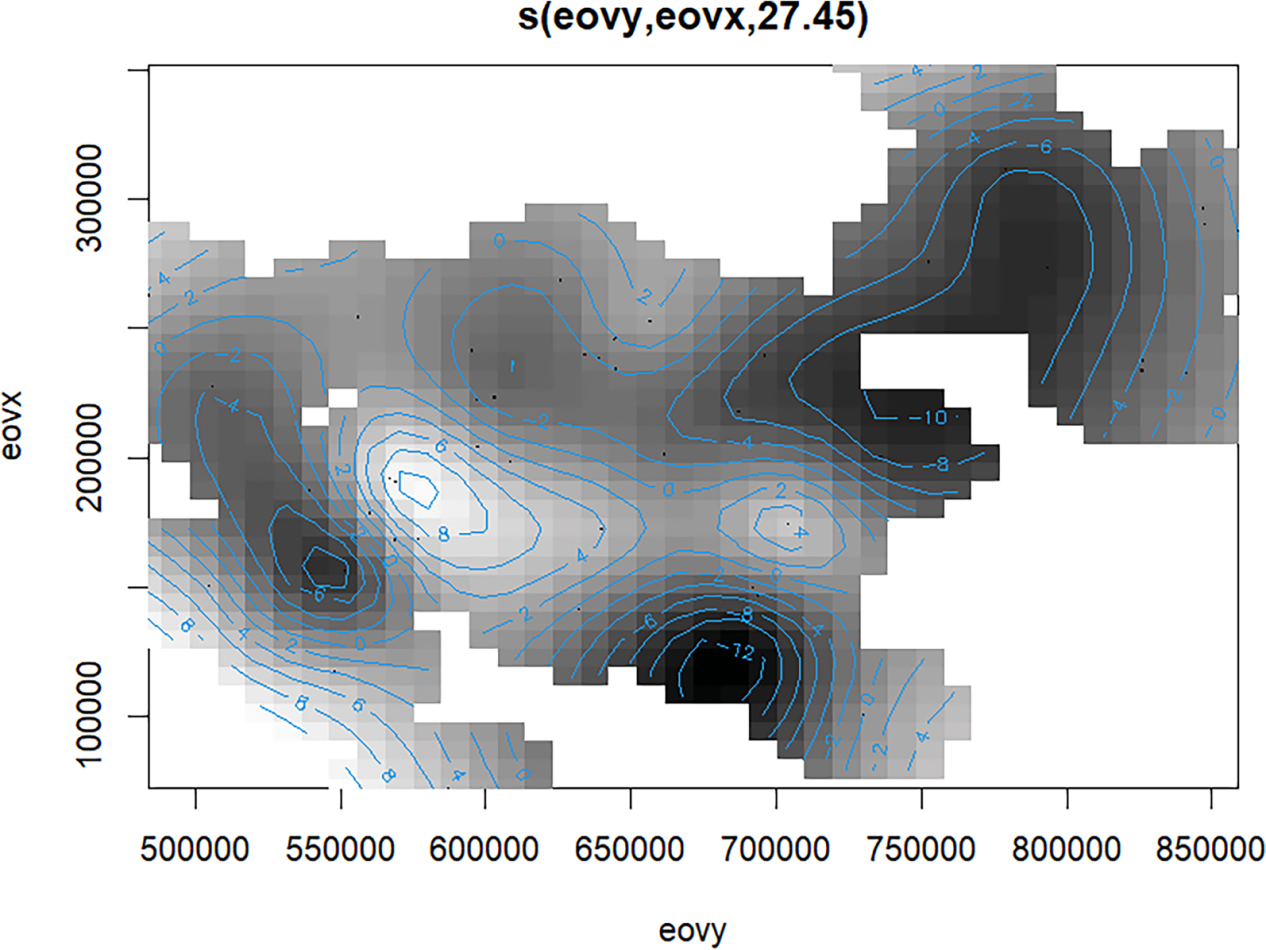
Contour plot depicting effects of location [*s(eovx and eovy)*] on density derived from the GAM model. The often concave, curved contour lines show a closely spaced pattern indicating a strong non-linear relationship between smooth terms of *eovx*, *eovy*. Their patterns identify certain regions where the density tends to increase (from contour line with zero to white areas with contour line with positive change up to 10) and regions where it tends to decrease (black areas with contour lines down to −12). Moreover, there are a few areas without any contour lines where the relationship between the smooth terms of *eovx*, *eovy* seems weak

**Fig. 4:**
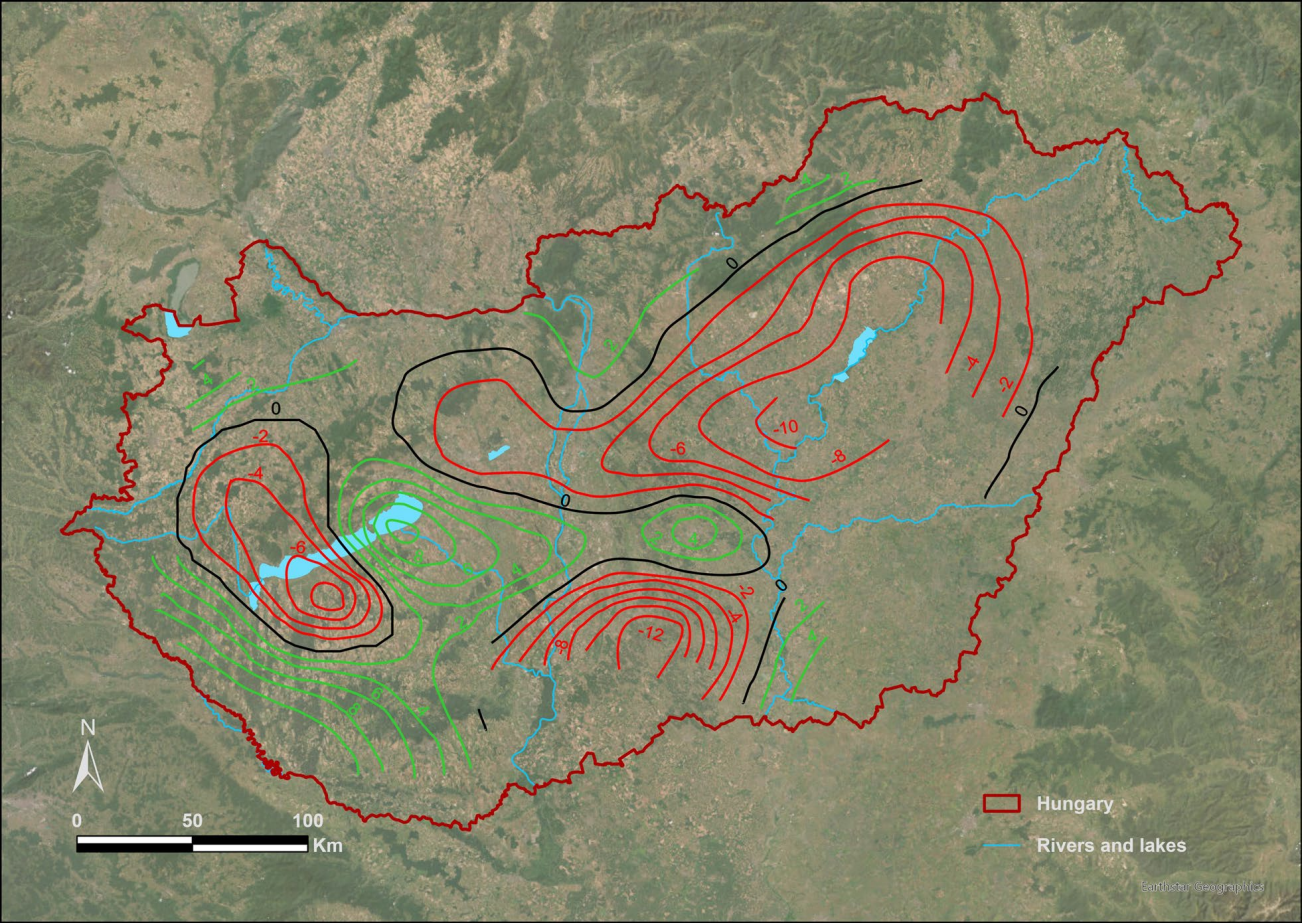
The georeferenced contour plot shows a dependence of population density of EGS colonies on location specific factors or a spatial effect. Green contour lines show positive, red contour lines show negative, black contour lines correspond to where population density is equal to the overall mean density. Hiatuses in the contour lines refer to lack or insufficient data points or the smooth spline surface is unstable

Since Fig. 3 shows the original contour map without any spatial reference, for easier interpretation we georeferenced the contour plot so that the spatial location of high or low counts observed was put in a real geographic place (Fig. 4).

Based on the information-theoretic approach, we identified five best-ranked models (from 512 models) (Supplementary Table 1.), which always contained predictors *Airfield*, smooth functions of *C3S*, *eovx/eovy*, and *year*, indicating those predictors importance for model plausibility. *C3S* corresponded to the monthly temperature of the wettest month in the active period. Cumulative Akaike weight values of these five models ranged from 0.062 to 0.022. The simplest (most parsimonious) model contained four predictors with a cumulative Akaike weight of 0.033: *Airfield*, s(*C3S*), s(*eovx, eovy*), and s(*year*). Sum of weights score (SW) for each term across all models showed the importance of these four predictors in the set of models considered: location (*eovx*, *eovy*, (SW = 1); *year* (SW = 1); *Airfield*, (SW = 0.76); *s(C3S)*, (SW = 0.74).

Moreover, regarding synchronised cyclicity or regular changes in counts of colonies, visual detection of changes in counts (Supplementary Fig. 1.) during the period of 19 years and within our dataset has not shown visual evidence to proceed with a statistical analysis to check for population cycles. In this respect, we followed the recommendations not to go for cycles if they are not detectable [81].

## Discussion

Our aim was to investigate the potential effects of various abiotic parameters representing land use type (either an airfield or any other) and weather features on the population density reflected by annual counts of active burrows of EGS in Hungary between 2000 and 2018. For weather variables, we evaluated the effects of a PCA‑derived subset of predictors consolidated from a more extensive set of climatic parameters. Moreover, we added the year of observation and geographic location that were expected to influence counts during active or hibernating (inactive) periods of EGS.

The final model derived from a sequential model selection approach included location (*eovx* and *eovy*) and *year* to affect density significantly. Those two predictors’ primary importance was also reflected in the information-theoretic approach. Considering the observation years, we found that EGS populations showed a decreasing trend (Fig. 2A). Moreover, there was no sign of a synchronised pattern or cycles (Supplementary Fig. 1) within that 19 years between 2000 and 2018; however, the currently studied observation period and our reduced dataset could have been simply and likely not long and large enough to detect these kinds of changes and population cycles particularly [82–84]. Rodent populations could have cycles of 3–5 (smaller size) or 9–11 years (larger size, such as *Spermophilus pygmaeus*), which shows that the current period of 19 years could not have been long enough to detect a longer cyclicity. Furthermore, additional climatic or anthropogenic factors could further dampen the natural pattern [85–87]. Consequently, further research need to focus on individual colonies with longer, consecutive data sets [16].

Lack of synchronisation among colonies, either cycles or non-cycling changes, experienced in this study could also mean that various non-climatic environmental variables, such as soil characteristics or their interconnection to other abiotic, landscape features, or density-dependent factors might have acted non-continuously or stochastically [88]. In other words, local, stochastic or chaotic, exogenous factors could have dominated population density changes [89–91].

Geographic location (*eovyx* and *eovy*) was found to be by far the main predictor of the change of counts in both approaches. While it was a well-defined variable in the model, location per se cannot give a clear explanation for counts change since there are numerous local factors and stressors [92–95] acting on colonies that could have positive or negative impact on counts and corresponding density of EGS populations. There is a large amount of evidence that supports the importance of local factors, for instance, ecological isolation [38] of colonies, microtopography (particularly undulating landscapes) [43, 96], agricultural or human activity including habitat destruction or abandonment of grassland management [53, 94, 97–101]. Nevertheless, the uneven spatial distribution of high or low counts observed illustrated by the contour plot (Fig. 4) closely corresponds to the spatial distribution of flood regions of Hungary.

Flood regions of rivers Danube and Tisza particularly, and Lake Balaton [102–104] overlap most with the lowest counts observed on the Great Hungarian Plain or west and northwest of Lake Balaton. River Tisza’s water catchment area and the Great Hungarian Plain is a typical low-lying grassland, with little elevation differences, exposing the entire area to increased flood and water excess hazards [105, 106]. An earlier study [35] has found, while investigating historical records of EGS populations, that the southeastern part of Hungary and Transtisza region have not had a large EGS population for centuries despite the species having been in the Carpathian Basin for at least 2000 years and the area could have provided seemingly adequate habitats for colonisation. That finding agrees with the current density map indicating that EGS colonies in regions with higher flood hazard show a more negative population trend compared to central (Danube-Tisza Interfluve, Kiskunság meso-region, Sand Ridges micro-region) or northwestern (Kisalföld macro-region) parts of the country [107], where flood hazard is lower because of more intensive aridification and soil conditions with favourable water management characteristics and structure[108]. Particularly, in the Kiskunság meso-region, which is considered the driest sand region of Hungary [63, 108], the Hungarian state ranger service has reported an increasing population trend and colonisation of new areas in the past 5 years (unpublished reports). Another piece of evidence for the increase in density and counts experienced within this study in the Kiskunság (Bács-Kiskun County) depicted by the contour plot is supported by recent data collected from the WildWatcher citizen science observational network program (Vadonleső Program, www.vadonlesoprogram.hu) in the last 10 years. The interactive webpage of the program enables citizens to record online and in real time the spatial location of EGS observations [109]. Data showed that there was a twofold increase in the number of observations between 2020 and 2025 (18 obs.) compared to 2015–2020 (9 obs.). To account for the varying sampling (observation) effort between these two periods, we used the case of the Northern white-breasted hedgehog (*Erinaceus roumanicus*) as a reference, whose presence has been continuously recorded since 2009 (beginning of the program) in large numbers and all over the country. In total, 54% of observations of hedgehog’s fell between 2015 and 2020 and 46% between 2020 and 2025 in Bács-Kiskun County (Kiskunság). We compared EGS’s presence data across the two time periods using hedgehog as a reference. We used the Chi-square test of independence to test if the observed changes in EGS presence are statistically significantly related to the changes in hedgehog’s presence [110]. By using that information, we could state that over the past five years significantly more EGS observations (twofold increase) have been recorded in the program than in the period between 2015 and 2020 (Chi^2^ = 8.138, df = 1, *p* = 0.0043). A positive effect of droughts (aridification) on the expansion of rodents has been found for prairie dogs [111] and other muroid rodents as well [112]. Kiskunság has been characterised by increased aridification, decreasing groundwater level, and more frequent droughts due to a warming climate, whereas other flat areas in the Great Hungarian Plain are more prone to riverine floods in early spring (March, April) or summer (June) [108] (C2W, C3W components in our model referred primarily to precipitation and temperature).

Another region with the lowest counts observed of ground squirrel burrows depicted by our contour plot overlaps with flash flood areas near Lake Balaton. Those hilly and low-mountainous areas at the northwestern edge of the lake are vulnerable to flash floods due to various topographic, soil physical, and hydrological factors [113]. Inundation of burrows can increase stress on ground dwelling animals like EGS in both active and inactive periods, which could trigger the activation of acute or general stress response [92, 93]. For instance, fast watering of burrows during the active period could cause hypothermia, pneumonia, and finally death of large number of animals. The torpor-like physiological response during watering of animals combined with lower temperatures have already been experienced in translocations during spring periods [114] or during the inundation of EGS colonies, near Budapest at Vöröskővár in 2007, in Esztergom in 2010 (https://www.origo.hu/tudomany/2011/01/tomeges-allatpusztulasok-legvezeteknek-utkozo-madarak-sok-eso-miatt-pusztulo-urgek) or in Biele Vody, Slovakia (2020) (https://en.syslinavinici.cz/news/washed-out-ground-squirrels-at-the-biele-vody-locality-slovakia), when torrential rain caused the death of hundreds of individuals. Following the rainfall, animals left their burrows and then stayed on the surface near the openings, more-or-less motionless, for several hours. Those events illustrated the sudden negative effect of inundation on population density, which in the long run could mean that population census and effective size could fluctuate more intensively and unexpectedly in these areas, increasing the risk of population collapses or sudden falls in the number of breeding animals in a colony. During hibernation or torpor-like physiological conditions, inundation of burrows can likely result in immediate drowning of animals and collapse of colonies.

For the predictor *Airfield*, which was also found to be an important predictor in both approaches, our results highlighted its effect on EGS population density. Sites managed as grassy airfields consistently supported higher densities than non‑airfield habitats, indicating that this land‑use type provides conditions favourable for the species. The most plausible explanation is that airfields combine several key ecological advantages: reduced human disturbance, lower predation pressure from birds of prey due to open visibility and restricted access, and maintenance of short grass. Because these management practices are essential for airfield operation, they can sustain a higher‑quality habitat than non-airfields, where these management practices are irregular or missing. Consequently, grassy airfields have become important refuges for ground squirrels in Hungary, offering stable habitat patches in landscapes where suitable grasslands have otherwise declined.

C2W, C3W, and C3S were the principal components that most consistently appeared to predict variation in EGS population counts. It is noteworthy that the traditional approach highlighted C2W, whereas the information‑theoretic framework suggested greater support for C3S. A response plot (Fig. 2. B, C) of those principal components from the best-fitting GAM model illustrated the impact of weather components on the counts. C2W showed high positive loading for *tphm* (loading = 0.89), indicating the effect of total monthly precipitation of the warmest month during hibernation. Other variables with a factor loading of 0.8 (loading_tp_ = 0.88; loading_tpcm_ = 0.81) corresponded also to precipitation, indicating that C2W could be best characterised by “precipitation during hibernation”. C3W was characterised by strong associations with *mthm* (loading = –0.69) and *mtwm* (loading = 0.82) indicating the effect of the mean monthly temperature of the warmest and wettest months during hibernation, though, with opposite signs. Regarding *C3S*, which had a strong association with *mtwm* (loading = 0.87), it indicated the positive effect of mean monthly temperature of the wettest month on the counts in the active period. To summarise these findings, they indicated that temperature and precipitation and probably their non-linear interaction may significantly affect density during both inactive (contributing to how animals survive hibernation) and active periods, likely via and in interaction with various factors, such as the quantity and composition of food resources or survival of offsprings [15, 30]. Another lesson to learn from the results is that weather variables have intensified importance on counts during the warmest and wettest months. Climate change will likely alter the amount and distribution of precipitation and increase temperature all year round in the Carpathian Basin, and it could change the time course and characteristics of the "wettest" and "warmest" months during hibernation, which will likely affect survival of EGS. Though the interaction of those components were not investigated, results supported that the changing trend of temperature and precipitation (components C2W, C3W, C3S) resulting in aridification (decreased risk of flooding) and temperature rise, which is predicted by climate models (finer-scale weather predictions), may positively affect survival during active and inactive periods [115, 116]. A recent study indicated the paramount importance of mean temperature of the wettest and driest periods, and diurnal temperature range on the distribution of EGS both for the southern, from Ukraine to Turkey, and northern, from the Czech Republic to Serbia, genetic lineages of the distribution area, which is in agreement with our results [117]. For a ground squirrel with similar size and ecology, the Columbian ground squirrel (*Spermophilus columbianus*), it was found [118] that delayed snowmelt and lower spring temperature (the opposite of our findings considering the predicted temperature change in Hungary) resulted in delayed emergence from hibernation. For another ground squirrel species (*S. pygmaeus*), increased top temperatures in February resulted in earlier emergence from hibernation and dispersal [119]. For *Urocitellus armatus*, juveniles’ and adults’ survival chances changed oppositely indicating that a warming climate could have both positive and negative effects on specimens of different age classes [120].

Ground squirrels show a wide range of plasticity and flexibility with their response to a changing climate; therefore it is difficult and would be unsubstantiated to identify general patterns and physiological, behavioural responses to climate change [121]. The above-mentioned studies found that some changing climate factors could affect mean fitness or population growth negatively due to limited access to food (snow cover) and limited time to fatten for next hibernation, however, other changes, such as increased spring temperatures were shown to increase earlier emergence from hibernation, and thus the time available to forage, which should have a positive effect on fitness. Although droughts may change soil and landscape properties negatively for EGS through increased hardness of the soil, less vegetation, etc., this factor seemed to influence population density positively according to our results. Consequently, moderate drying of this region could be considered promising for species conservation.

Our dataset and corresponding model selection have come to these conclusions, nevertheless, we need to keep in mind our constrained dataset and draw careful conclusions from the results. For instance, the yearly decreasing trend of counts, stretches of zeros for consecutive years, possible autocorrelation, etc. indicated a smooth term for the effect of year on counts. A future study on a more complete dataset may identify different factors or relationships between the response variable and predictors (linear relationships), therefore those factors or relationships ought to be reconsidered in future works to have a clearer picture on the driving factors behind the change of population density of EGS.

Our model did not investigate the effect of other abiotic characteristics, such as soil, landscape, isolation, etc. and the complex interaction between those factors including with weather parameters. For instance, EGSs living in sandy soil that drains well would not be impacted as strongly as those in clay-rich soil by precipitation and flooding events. Very high water infiltration rate (IR), permeability (P), hydraulic conductivity (HC), and low field capacity (FC) and very poor water retention (WR) characterise the Kiskunság Sand Ridge, and moderately high IR, P, HC, FC, and WR characterise the Kisalföld or central, Transdanubian micro-regions, where yearly counts observed was increasing [122]; Supplementary Fig. 3). In simple terms, sandy soils could provide better digging options and water management characteristics for EGS, as long as, it maintains its structural integrity for burrows. All these rather suppositions indicate the potential importance of complex interactions between soil conditions, temperature, precipitation and life history traits, which should further be investigated.

We did not study interactions between predictors (the effect of C2W, C3W, C3S), however, high temperatures combined with increased precipitation during the wettest month particularly (melted snow) could disturb hibernation [27, 123]. Depending on the soil conditions locally, rain or melted snow can either inundate the animals, decrease the thermal insulation capacity of the soil or send wrong signals to the animals about the surface temperatures and conditions. Inundation’s negative effect is obvious, but if it is less severe, it still could mislead animals into an altered hypothermia-normothermia cycle with shorter periods of hypothermia and longer periods of normothermia episodes. For other hibernating species, such as the Northern white-breasted hedgehog (*Erinaceus roumanicus*) or the Eversmann’s hamster (*Allocricetulus eversmanni*), physiological processes (e.g. cooling and warming phases, length of hypothermia, etc.) are in correlation with the ambient temperature [124, 125]. Their extended normothermic episodes, because of increased ambient temperatures, could lead to increased energy consumption, and eventually smaller body mass or hibernation starvation. That finding is independent from the fact that animals return to hypothermia after those normothermic episodes. Though, observations of Murie and Michener [126] showed that ground squirrels stayed in hibernation despite a “January thaw”, nonetheless, it did not mean that their energy consumption had not been disturbed during unexpectedly warmer and wetter periods in hibernation. Although the hibernation chamber is generally well buffered from changing ambient conditions due to its depth, however, both external and internal stimuli could still influence the hibernation cycle and hibernators could react to altered ambient temperature and precipitation patterns combined or could sense other environmental parameters, such as soil moisture content [125, 127, 128].

## Conclusions

If we sum our results for a practical conclusion, it is important to underscore that the effect of weather factors was dwarfed by spatial effect or other location-specific factors, such as local protection through land use type or ownership (airfield, state owned lands) against predation or accidental or intentional hunting. Those local factors could be altered more easily than climate related factors. Location-specific factors, including resource availably, biotic and abiotic interactions, habitat loss, etc. can overrun the effect of macroecological processes, decreasing or increasing population or demographic variability [129, 130]. Location-specific variation in density has implications of paramount importance for any action plan for the species conservation and highlights the importance of local management decisions and actions aiming at strengthening the populations of ground dwelling sciurids by animal translocations or managing their habitats to support those local populations by, for example, maintaining short grass at EGS habitats. A large-scale management action plan (LIFE Nature proposal called *Citellus*Life, No.101202727) has recently gotten support from the European Union that is a practical application of this conservation approach. Complex interactions between driving factors, their spatial variation in their effect, etc. all make local studies critically important to first identify those local drivers and then to apply relevant actions to deal with them for species conservation [129].

## Supplementary Information

Additional file 1

Additional file 2

Additional file 3

Additional file 4

Additional file 5

## Data Availability

Data will be provided and archived (for at least 5 years) at the https://owncloud.atk.hu/index.php/s/HMLOKNIKYjdqyU8 upon acceptance of the manuscript for publication in the Frontiers in Zoology.

## Abbreviations

IUCN: International union for conservation of nature (https://iucn.org/)
EGS: European ground squirrel (Spermophilus citellus)
FORESEE: The FORESEE database, open database for climate change-related impact studies in central Europe) (https://nimbus.elte.hu/FORESEE/)
DOSoReMI: Digital, optimised, soil related maps and information in Hungary (https://dosoremi.hu/en/)
GAM: Generalised additive model
PCA: Principal component analysis
PC: Principal component
*C1S, C2S*,…, *C3W*: Principal components from PCA analysis; S or W refers to summer (active) or winter (hibernating) periods of ground squirrels
*mtss*: Mean monthly temperature between sunrise and sunset (°C)
*mt24h*: Mean monthly temp between 0 and 24 h (°C)
*tp*: Total monthly precipitation (mm)
*mthm*: Mean monthly temperature of the warmest month (°C)
*tphm*: Total monthly precipitation of the warmest month (mm)
*mtcm*: Mean monthly temperature of the coldest month (°C)
*tpcm*: Total monthly precipitation of the coldest month (mm)
*mtwm*: Mean monthly temperature of the wettest month (°C)
*GCV*: Generalised cross-validation score
*loccode*: Colony ID, an individual colony code
*EDF*: Effective degrees of freedom
