# Supplementary material for: What drives population fluctuations of European ground squirrels in Hungary?
Source: Front Zool. 2026 Apr 8;23:17. doi: 10.1186/s12983-026-00608-3 (PMC13130411; doi:10.1186/s12983-026-00608-3)
Supplement: Supplementary file 2 — Additional file 2 [file 12983_2026_608_MOESM2_ESM.pdf]

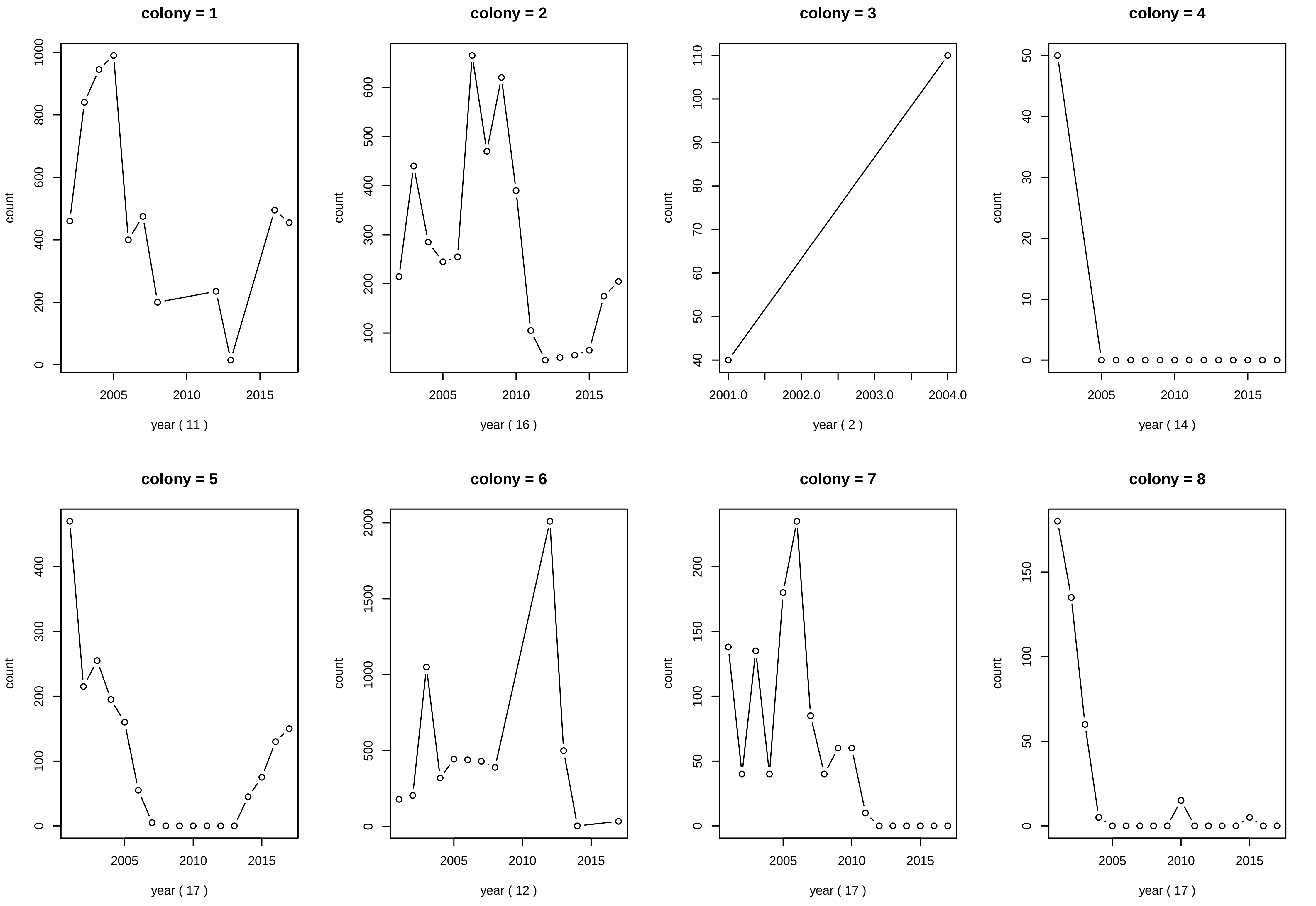
colony = 1
count
0
200
400
600
800
1000
2005
2010
2015
year (11)
colony = 2
count
100
200
300
400
500
600
2005
2010
2015
year (16)
colony = 3
count
40
50
60
70
80
90
100
110
2001.0
2002.0
2003.0
2004.0
year (2)
colony = 4
count
0
10
20
30
40
50
2005
2010
2015
year (14)
colony = 5
count
0
100
200
300
400
2005
2010
2015
year (17)
colony = 6
count
0
500
1000
1500
2000
2005
2010
2015
year (12)
colony = 7
count
0
50
100
150
200
2005
2010
2015
year (17)
colony = 8
count
0
50
100
150
2005
2010
2015
year (17)

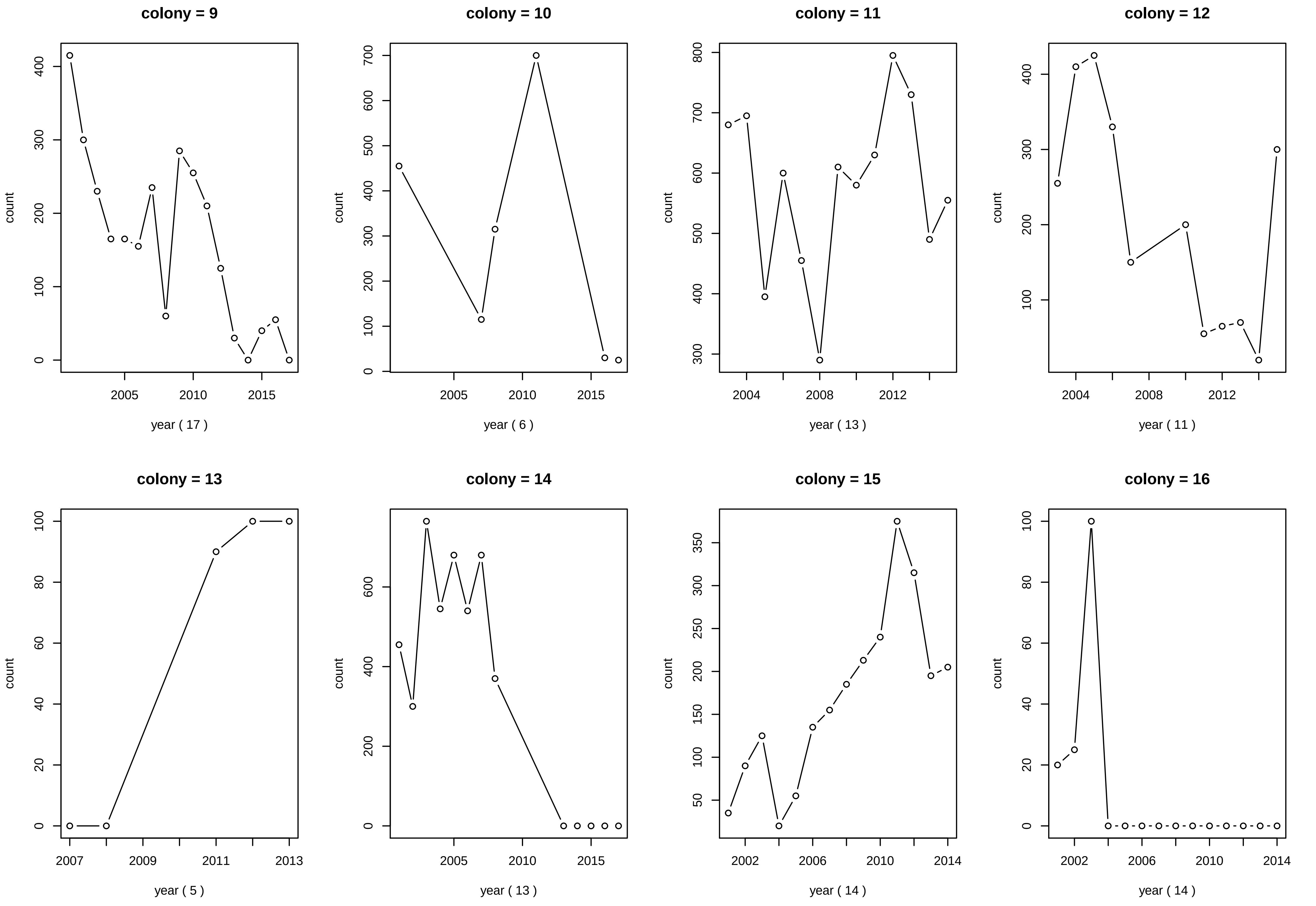

colony = 9
count
year (17)
colony = 10
count
year (6)
colony = 11
count
year (13)
colony = 12
count
year (11)
colony = 13
count
year (5)
colony = 14
count
year (13)
colony = 15
count
year (14)
colony = 16
count
year (14)

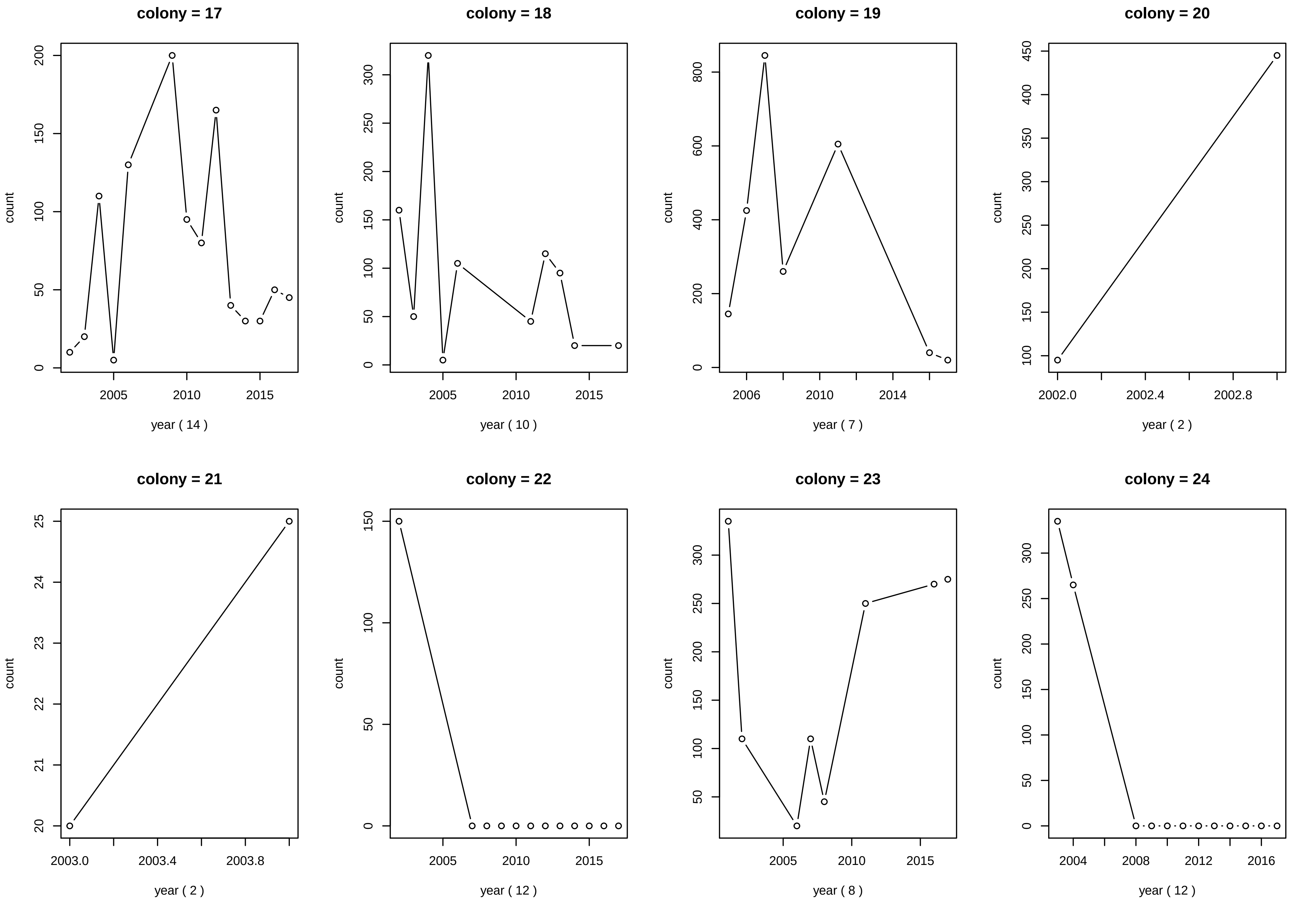
colony = 17
count
0
50
100
150
200
2005
2010
2015
year (14)
colony = 18
count
0
50
100
150
200
250
300
2005
2010
2015
year (10)
colony = 19
count
0
200
400
600
800
2006
2010
2014
year (7)
colony = 20
count
100
150
200
250
300
350
400
450
2002.0
2002.4
2002.8
year (2)
colony = 21
count
20
21
22
23
24
25
2003.0
2003.4
2003.8
year (2)
colony = 22
count
0
50
100
150
2005
2010
2015
year (12)
colony = 23
count
50
100
150
200
250
300
2005
2010
2015
year (8)
colony = 24
count
0
50
100
150
200
250
300
2004
2008
2012
2016
year (12)

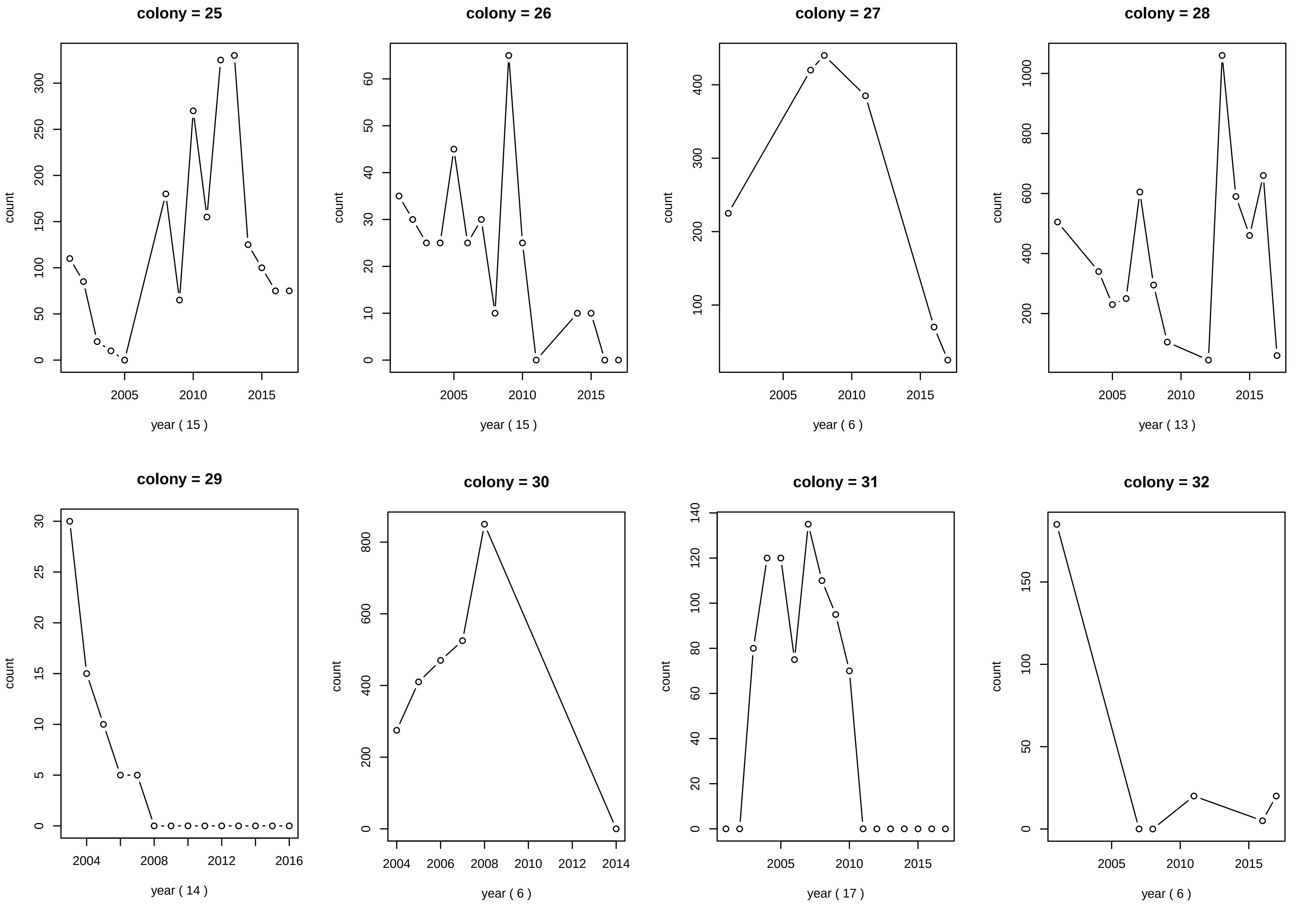

colony = 25
colony = 26
colony = 27
colony = 28
colony = 29
colony = 30
colony = 31
colony = 32
count
year (15)
year (15)
year (6)
year (13)
year (14)
year (6)
year (17)
year (6)

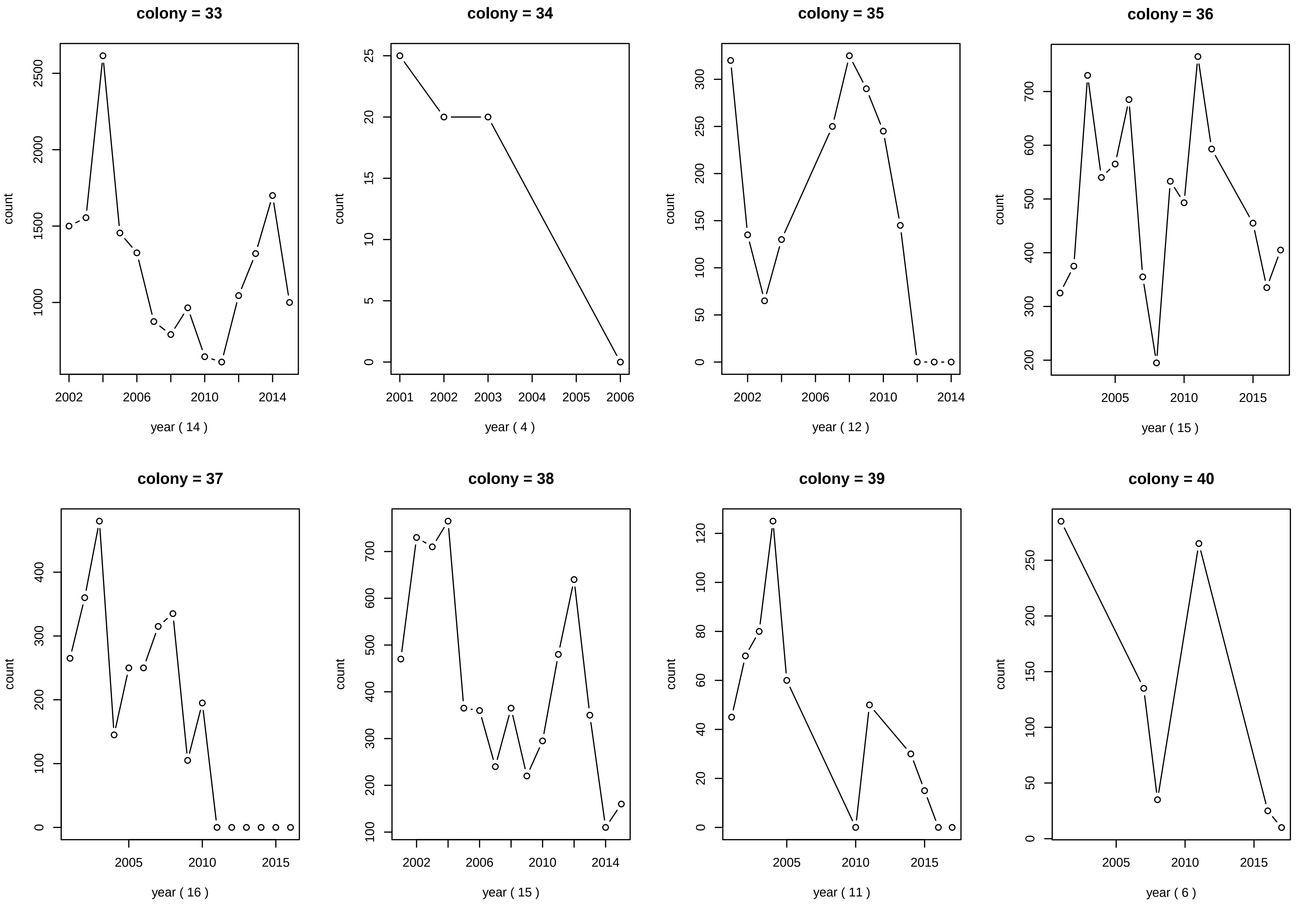

colony = 33
count
1000
1500
2000
2500
2002
2006
2010
2014
year (14)
colony = 34
count
0
5
10
15
20
25
2001
2002
2003
2004
2005
2006
year (4)
colony = 35
count
0
50
100
150
200
250
300
2002
2006
2010
2014
year (12)
colony = 36
count
200
300
400
500
600
700
2005
2010
2015
year (15)
colony = 37
count
0
100
200
300
400
2005
2010
2015
year (16)
colony = 38
count
100
200
300
400
500
600
700
2002
2006
2010
2014
year (15)
colony = 39
count
0
20
40
60
80
100
120
2005
2010
2015
year (11)
colony = 40
count
0
50
100
150
200
250
2005
2010
2015
year (6)

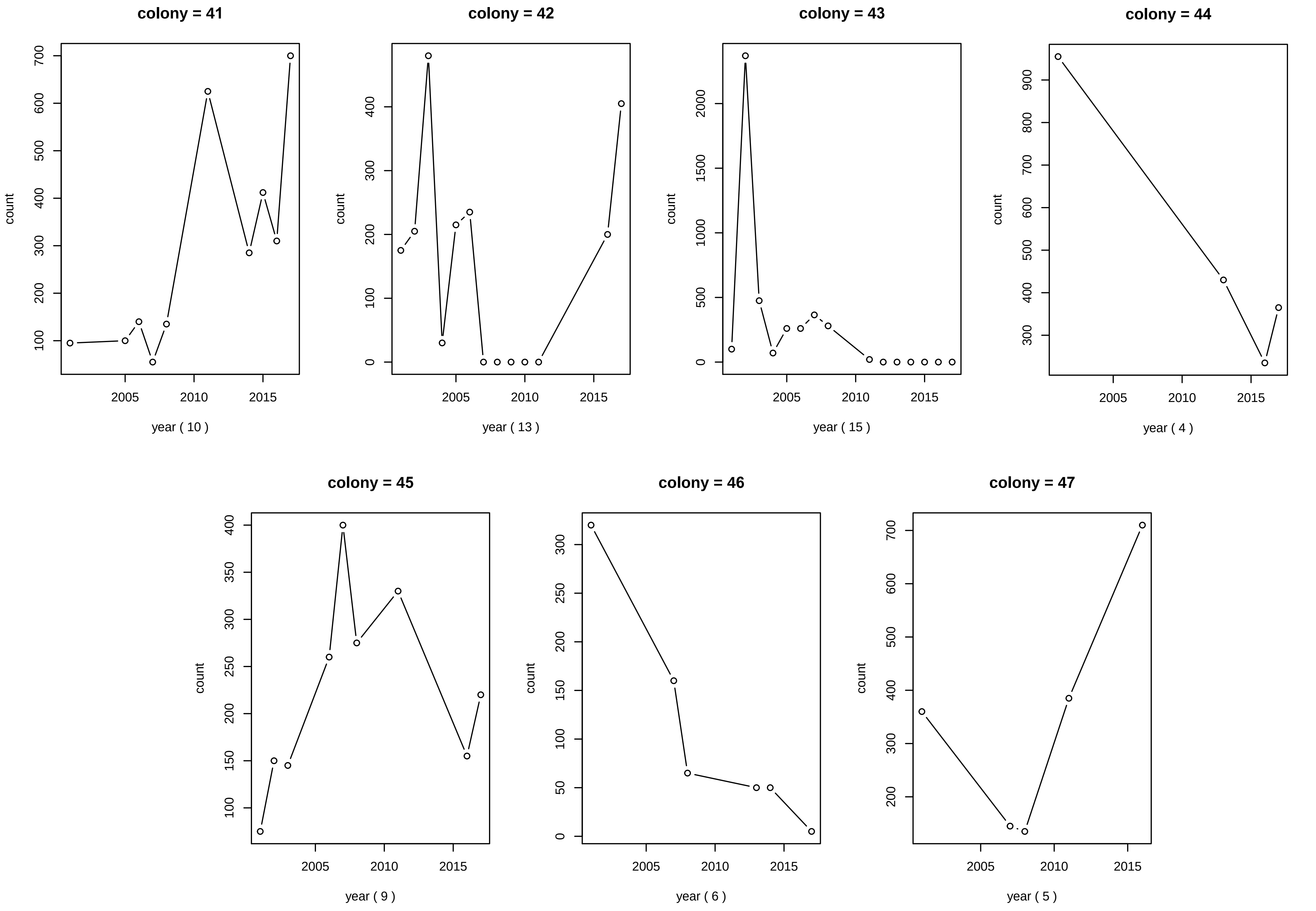

colony = 41
count
year (10)
colony = 42
count
year (13)
colony = 43
count
year (15)
colony = 44
count
year (4)
colony = 45
count
year (9)
colony = 46
count
year (6)
colony = 47
count
year (5)
