# Supplementary figures and images for: What drives population fluctuations of European ground squirrels in Hungary?
Source: Front Zool. 2026 Apr 8;23:17. doi: 10.1186/s12983-026-00608-3 (PMC13130411; doi:10.1186/s12983-026-00608-3)

Adjusted R<sup>2</sup>

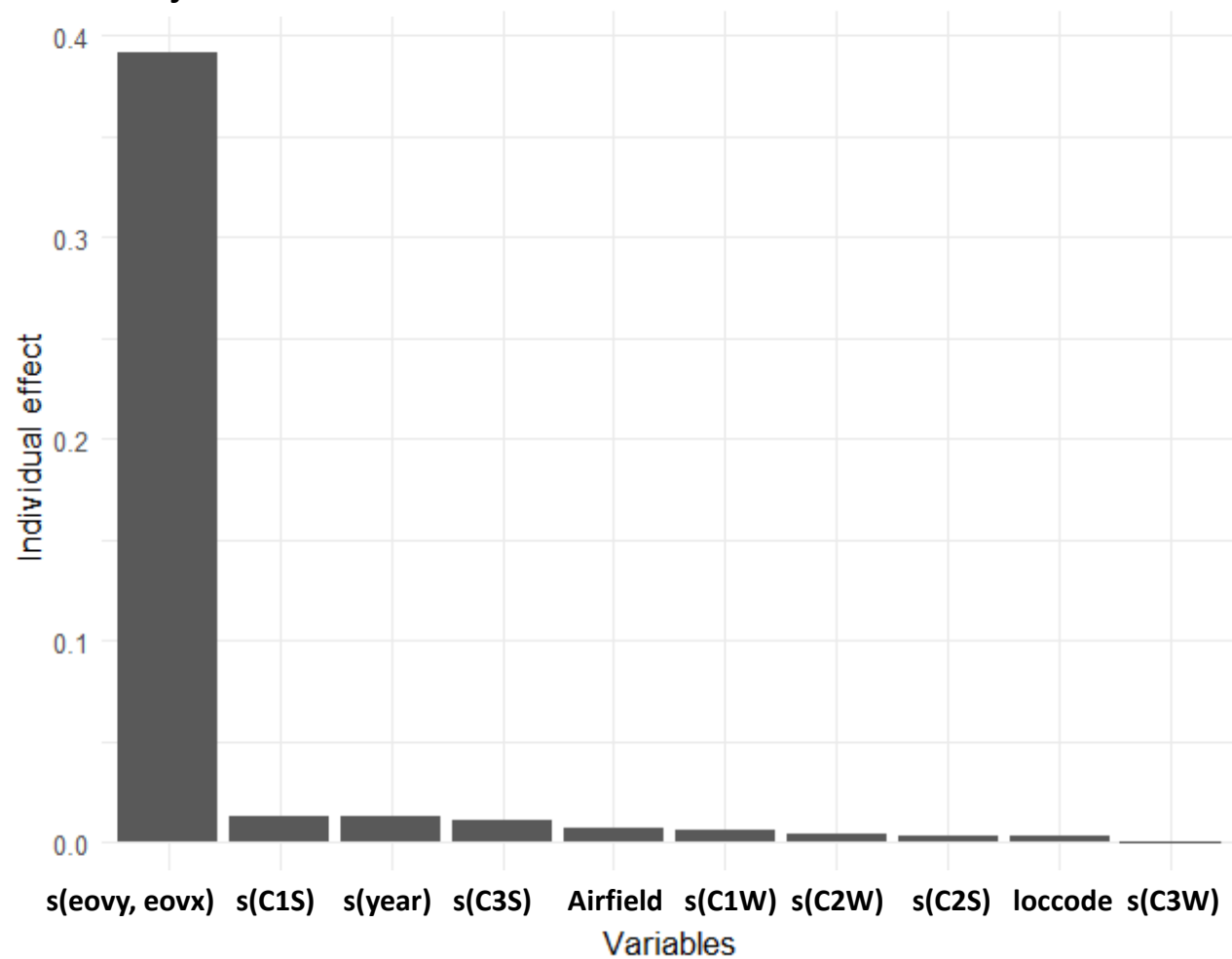

Supplement: Supplementary file 3 — Additional file 3 [file 12983_2026_608_MOESM3_ESM.pdf]

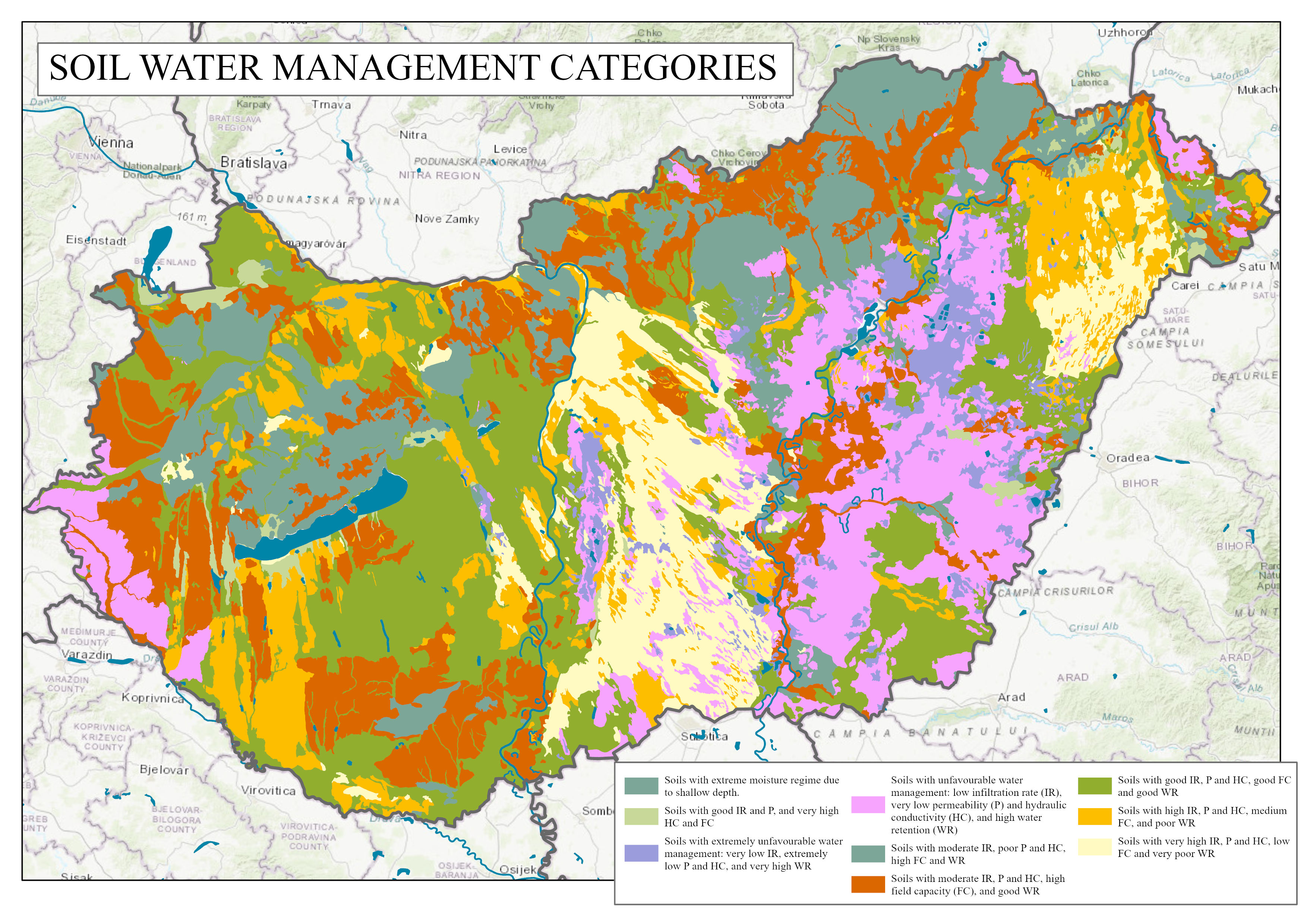

Supplement: Supplementary file 4 — Additional file 4 [file 12983_2026_608_MOESM4_ESM.jpg]
