# Supplementary material for: What drives population fluctuations of European ground squirrels in Hungary?
Source: Front Zool. 2026 Apr 8;23:17. doi: 10.1186/s12983-026-00608-3 (PMC13130411; doi:10.1186/s12983-026-00608-3)
Supplement: Supplementary file 5 — Additional file 5 [file 12983_2026_608_MOESM5_ESM.docx]

**Supplementary materials**

**Supplementary Figure 1.** Density changes of individual EGS colonies between years.

**Supplementary Figure 2.** Adjusted R^2^ explained of each predictor in the model. The effect of location *s*(*eovx* and *eovy*) clearly dominates the explanatory power of the model.

**Supplementary Figure 3.** Soil water management categories of Hungarian soils and the map of soil water properties (1:100000) by Várallyay et al., 1980.

**Supplementary Table 1.** Information-theoretic model comparisons in rank order of the five most parsimonious models. In addition to predictors in the models, Akaike Information Criterion (AICc), Cumulative Akaike weights, Sum of (Akaike) weights for each predictor, AIC differences are presented in the table. The number of models containing each predictor was 512.

| Model | AICc | Δ_i_ | Cumulative Akaike weight | df | Arf | s(C1S) | s(C1W) | s(C2S) | s(C2W) | s(C3S) | s(C3W) | s(eovy,eovx) | s(lcc,"re") | s(year) |
| --- | --- | --- | --- | --- | --- | --- | --- | --- | --- | --- | --- | --- | --- | --- |
| 930 | 2576.6 | 0.00 | 0.062 | 39.79 | **+** |  |  |  |  | **+** |  | **+** | **+** | **+** |
| 934 | 2577.5 | 0.93 | 0.039 | 41.76 | **+** |  | **+** |  |  | **+** |  | **+** | **+** | **+** |
| 932 | 2577.9 | 1.30 | 0.033 | 40.67 | **+** | **+** |  |  |  | **+** |  | **+** | **+** | **+** |
| 674 | 2577.9 | 1.30 | 0.033 | 39.32 | **+** |  |  |  |  | **+** |  | **+** |  | **+** |
| 946 | 2578.6 | 2.04 | 0.022 | 40.70 | **+** |  |  |  | **+** | **+** |  | **+** | **+** | **+** |
| Sum of  weights for predictor |  |  |  |  | 0.76 | 0.39 | 0.49 | 0.27 | 0.37 | 0.74 | 0.32 | 1 | 0.62 | 1 |
